# Supplementary material for: Inflammatory factors and risk of lung adenocarcinoma: a Mendelian randomization study mediated by blood metabolites
Source: Front Endocrinol (Lausanne). 2024 Aug 27;15:1446863. doi: 10.3389/fendo.2024.1446863 (PMC11384989; doi:10.3389/fendo.2024.1446863)
Supplement: Supplementary file 1 [file DataSheet1.zip › supplementary files/S2/GCST90200792/forest.pdf]

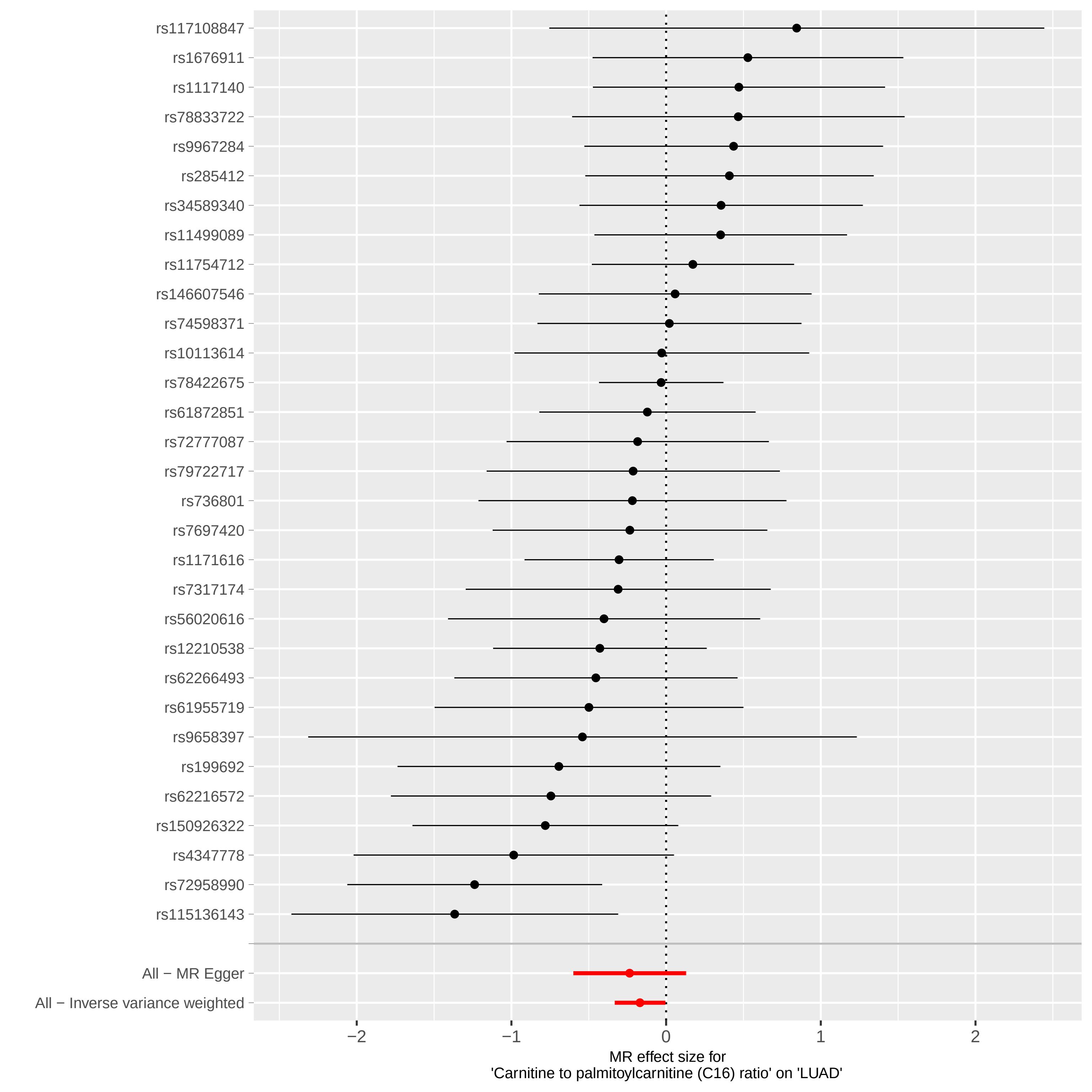

rs117108847
rs1676911
rs1117140
rs78833722
rs9967284
rs285412
rs34589340
rs11499089
rs11754712
rs146607546
rs74598371
rs10113614
rs78422675
rs61872851
rs72777087
rs79722717
rs736801
rs7697420
rs1171616
rs7317174
rs56020616
rs12210538
rs62266493
rs61955719
rs9658397
rs199692
rs62216572
rs150926322
rs4347778
rs72958990
rs115136143
All − MR Egger
All − Inverse variance weighted
−2
−1
0
1
2
MR effect size for
'Carnitine to palmitoylcarnitine (C16) ratio' on 'LUAD'
